# Follicular Lymphoma: Current Therapeutic Landscape and Future Prospects

**DOI:** 10.1002/hon.70070

**Published:** 2025-06-15

**Authors:** Emil Kumar, Jessica Okosun

**Affiliations:** ^1^ Department of Haematology King's College Hospital London UK; ^2^ Centre for Haemato‐Oncology Barts Cancer Institute Queen Mary University of London London UK

**Keywords:** biomarkers, bispecific antibodies, CAR‐T, follicular lymphoma, immunotherapy, minimal residual disease

## Abstract

Significant strides have been made in the treatment of follicular lymphoma, leading to improvements in long‐term patient outcomes. However, the disease's heterogeneity presents challenges in selecting the optimal therapy at each stage of treatment. The expanding array of therapeutic options introduces new complexities, including making the right initial choice, sequencing treatments effectively, and redefining treatment goals. As the landscape evolves, there is a growing need to shift toward precision‐based treatment decisions, potentially guided by underlying disease biology. Here, we explore recent advancements in both upfront and relapsed/refractory treatment strategies, addressing considerations in therapy selection, and the current progress toward precision approaches with its potential to enhance decision‐making.

## 1 Introduction

Follicular lymphoma (FL) is the second most common lymphoma subtype. Clinical behavior spans from asymptomatic patients, most not requiring therapy, to those with high‐risk disease and a poorer prognosis than typical FL patients. Recent clinical advances include novel immunotherapy options such as chimeric antigen receptor T cells (CAR‐T) and CD3 × CD20 bispecific antibodies (BsAb) for relapsed/refractory (R/R) FL patients. Unmet needs persist, especially for high‐risk patients experiencing early disease progression within 24 months of first‐line immunochemotherapy (“POD24”) or histologic transformation (HT). The biological underpinnings of FL are also better understood [1]. Bridging these bench insights into biology‐informed approaches is high on the research agenda. Equally important is balancing treatment efficacy with toxicity reduction, while ensuring personalized, cost‐effective care.

This review highlights key treatment updates for newly diagnosed and R/R FL patients, associated challenges, and where we are with improving precision oncology.

## 2 Getting It Right First Time: Dilemmas in Initial FL Management

### 2.1 Low Tumor Burden FL: Watch and Wait?

For asymptomatic low tumor burden (LTB) FL, the need for initial therapy is still strongly debated. Long‐term watch and wait (W&W) studies show single‐agent rituximab induction (RI) +/− maintenance rituximab (RM) extends median time to next treatment (W&W: 2.7 years, RI: 9.9 years vs. RI + RM: not reached) without compromising next treatment efficacy [2, 3]. The RESORT study, where LTB patients had RM after RI or rituximab‐retreatment at progression corroborates this, with 63%–73% of patients remaining cytotoxic‐free at 7 years [4, 5]. Emotional well‐being improved significantly with RI + RM compared to W&W, supporting an health‐related quality of life (HrQoL)‐based rationale for early treatment in select patients [6]. Equally, risks associated with rituximab‐induced B‐cell depletion, including impaired antiviral immunity, reduced vaccine responsiveness, hypogammaglobulinemia, require careful consideration. In the absence of an overall survival (OS) benefit, the occurrence of spontaneous regression in some patients and with nearly one‐third of W&W patients avoiding first‐line treatment even after 10 years [2, 3], all serve as crucial reminders that more is not necessarily better for every patient. This underscores the importance of considering toxicity, HrQoL factors, and the merits of identifying biomarkers for long‐term non‐progression.

### 2.2 Advanced Stage Symptomatic, High Tumor Burden FL

Most practice guidelines recommend either rituximab or obinutuzumab with chemotherapy for the treatment of symptomatic high tumor burden (HTB) FL [7, 8]. Importantly, one‐third of such patients treated with rituximab monotherapy remain treatment‐free at 10 years [9].

First‐line immunochemotherapy achieves excellent long‐term outcomes, for example, 10‐year progression‐free survival (PFS) at 35%–51% and OS at 80% [10], indicating the likelihood of a “functional cure” for many patients. Final analysis of the GALLIUM trial continues to confirm a PFS advantage for obinutuzumab (O)‐chemotherapy over rituximab (R)‐chemotherapy (7‐year PFS: 63% vs. 56%), though without OS benefit [11, 12]. Minimal residual disease (MRD) status in this trial, based on detectable t(14;18) translocation or clonal immunoglobulin heavy or light chain rearrangements, has a demonstrable association with outcomes [13]. Markedly lower rates of MRD negativity were seen in R‐CVP and R‐CHOP‐treated patients relative to R‐bendamustine, however, this was largely overcome by obinutuzumab combinations. Concerns about infectious toxicities with obinutuzumab, especially with bendamustine during maintenance [11], have limited its use in older or co‐morbid patients, with O‐CVP or R‐bendamustine often preferred. Since no anti‐CD20 antibody‐chemotherapy backbone has shown an OS benefit, the choice remains nuanced, influenced by patient age, fitness, and clinician preference.

Chemotherapy‐free approaches, such as R2 (lenalidomide‐rituximab), showed comparable 6‐year PFS and OS to R‐chemotherapy in the RELEVANCE study, with distinct toxicities associated with lenalidomide including rash and gastrointestinal disturbance [14]. Secondary primary malignancy (SPM) and transformation risk remains comparable.

Despite rituximab maintenance improving PFS [10], adoption of this approach widely varies, particularly with its prolonged B‐cell depletion and lack of OS benefit, prompting response‐adapted strategies to be considered (*discussed later*).

Due to promising efficacy of BsAbs (both CD3 × CD20 and CD3 × CD19), these are being evaluated as initial therapies in several ongoing randomized trials (Table 1).

**TABLE 1:** Selected ongoing BsAbs trials for untreated advanced stage high tumor burden FL patients.

Trial/NCT	Phase	Patient group	Planned enrollment	Experimental arm	Standard arm	Primary endpoint
EPCORE FL2 (NCT06191744)	III	1L	1080	Epcoritamab + R^2^, followed by epcoritamab maintenance if CR or PR	R^2^ or CIT (O/R‐CHOP or O/R‐B) + maintenance	CR30
						PFS
MorningLyte (NCT06284122)	III	1L (FLIPI 2–5)	790	Mosunetuzumab + lenalidomide	CIT (O/R‐CHOP or O/R‐B) + maintenance	PFS
OLYMPIA 1 (NCT06091254)	III	1L	446	Odronextamab, followed by odronextamab maintenance if CR or PR	CIT (R‐CHOP/CVP/B) + maintenance	CR30
SOUNDTRACK‐F1 (NCT06549595)	III	1L	1005	AZD0486 + R	CIT (R‐CHOP/CVP/B) + maintenance	PFS

Abbreviations: 1L = first line; AZD0486 = CD19 × CD3 BsAb; B = bendamustine; CHOP = cyclophosphamide, doxorubicin, vincristine, prednisolone; CIT = chemo‐immunotherapy; CR = complete response; CR30 = complete response at 30 months; CVP = cyclophosphamide, vincristine, prednisolone; FLIPI = follicular lymphoma international prognostic index; O = obinutuzumab; PFS = progression free survival; PR = partial response; R = rituximab; R^2^ = rituximab and lenalidomide.

### 2.3 POD24: Defining the Problem to Focus the Solution

POD24, affecting 10%–20% of patients, is associated with poor OS [15, 16, 17]. There is an increasing appreciation of the heterogeneity within POD24 cohorts. Early progression events are enriched for HT (POD24_HT), although the reported incidence as a proportion of all POD24 varies widely [18, 19], underlining our recommendation to always re‐biopsy at progression, in both clinical trial and real‐world scenarios. Recent retrospective analysis from the LEO consortium of real‐world evidence (CReWE) demonstrated particularly poor outcomes for POD24_HT, with 5‐year OS as low as 31%, whereas biopsy‐proven indolent FL POD24 (POD24_FL) exhibited better survival (71%) [20]. In one series, POD24_FL patients with low‐intermediate FLIPI had comparable OS to non‐POD24 patients [19].

The ability of maintenance rituximab, obinutuzumab induction, or bendamustine‐based induction to lower early progression rates without affecting OS or HT rate [10, 12, 18] suggests that not all POD24 cases are equal, with a subset remaining resistant to current treatments.

Autologous stem cell transplantation (ASCT) may offer OS benefits in POD24 cases, though randomized data are lacking [21, 22]. Clinical trials, such as SWOG1608, are assessing alternative approaches.

## 3 Treatment of Relapsed, Refractory FL: Spoilt for Choice?

The area of greatest therapeutic strides has been for R/R FL patients with autologous T cell‐harnessing immunotherapies: CAR‐T (Table 2) [23, 24, 25, 26, 27] and BsAbs (Table 3) [28, 29, 30, 31]. Since 2021, three CAR‐T constructs—axicabtagene‐ciloleucel (axi‐cel), tisagenlecleucel (tisa‐cel), and lisocabtagene maraleucel (liso‐cel)—and three CD3 × CD20 BsAbs—mosunetuzumab, epcoritamab, and odronextamab—have gained regulatory approval in Europe and/or US as options for third line and later.

**TABLE 2:** Summary of efficacy and safety data for CAR‐T in relapsed‐refractory FL.

CAR‐T product	Trial	Phase	Prior lines	No. of patients	ORR	CR	Median PFS	Median DoR	Median OS	CRS (gd ≥ 3)	ICANS (gd ≥ 3)
Axi‐cel	ZUMA‐5 [23, 24]	2	≥ 2	127	94%	79%	57.3 months	60.4 months	Not reached	78% (6%)	56% (18%)
Tisa‐cel	ELARA [25, 26]	2	≥ 2	97	86%	68%	53.3 months	74% at 12 months	Not reached	49% (0%)	23% (1%)
Liso‐cel	TRANSCEND [27]	2	≥ 2	101	97%	94%	73% at 24 months	82% at 12 months	Not reached	58% (1%)	15% (2%)

Abbreviations: CR = complete response rate; CRS = cytokine release syndrome; DoR = duration of response; gd = grade; ICANS = immune effector cell‐associated neurotoxicity syndrome; ORR = overall response rate; OS = overall survival; PFS = progression‐free survival.

**TABLE 3:** Summary of efficacy and safety data for BsAbs in relapsed‐refractory FL.

BsAbs	Prior lines	No. of FL patients	Delivery	ORR	CR	Median PFS	CRS gd 3–4	ICANS gd 3–4
Mosunetuzumab [28, 29]	≥ 2	90	IV	78%	60%	24 months	1%	0%
			21‐day cycles					
			Fixed duration					
Odronextamab [30]	≥ 2	128	IV	80%	73%	20.7 months	2%	3%
			28‐day cycles					
			Continuous until PD					
Epcoritamab [31]	≥ 2	128	SC	82%	62%	15.4 months	2%	0%
			28‐day cycles					
			Continuous until PD					

Abbreviations: CR = complete response rate; CRS = cytokine release syndrome; gd = grade; ICANS = immune effector cell‐associated neurotoxicity syndrome; IV = intravenous; ORR = overall response rate; PD = progressive disease; PFS = progression‐free survival; SC = subcutaneous.

### 3.1 CAR‐T

CD19‐directed CAR‐T therapies are highly effective in B‐cell lymphomas but present unique toxicities, including early cytokine release syndrome (CRS) and immune effector cell‐associated neurotoxicity syndrome (ICANS), and later sequelae including infections, cytopenias, and rare SPMs. In large B‐cell lymphomas (LBCL), infections are the leading cause of non‐relapse mortality (NRM) post CAR‐T [32], with half of infection‐related deaths due to COVID‐19 in one study [33]. SPMs occur in ∼15% at 5 years, comparable to historical lymphoma controls [34]. Despite early concerns, secondary T cell lymphomas (TCL) are rare [34, 35].

Data from the pivotal axi‐cel and tisa‐cel studies show high response rates in R/R FL [23, 25]. Attainment of CR appears crucial for durable responses, although this remains lower for patients with POD24. Recent updated analyses indicate that ∼50% of patients remain in long‐term remission [24, 26]. Whether these durable responses equate to potential cure is unclear. Liso‐cel (TRANSCEND FL) also demonstrates remarkable efficacy, even for POD24 patients [27]. Higher grade CRS and ICANS rates (≥ gd 3) are generally more common with axi‐cel (CD28 co‐stimulation) than both tisa‐cel and liso‐cel (4‐1BB co‐stimulation). Lower toxicity rates in tisa‐cel and liso‐cel, along with increased experience in managing these toxicities are enhancing the feasibility for outpatient delivery.

### 3.2 Bispecific Antibodies

Single‐agent BsAbs, combining CD20 targeting with CD3‐mediated T‐cell engagement demonstrate promising efficacy in 3L+ with manageable toxicity [28, 29, 30, 31]. Optimized dose escalation and steroid prophylaxis allow outpatient delivery with low rates of high‐grade CRS and ICANS.

Of the 3 BsAbs, mosunetuzumab is given as fixed duration intravenous (IV) therapy while subcutaneous epcoritamab and IV odronextamab are continuous until intolerance or disease progression. Overall response rates (ORR) are in the region of 80% with a median PFS of 15–24 months (Table 3). Compared to CAR‐T, high grade CRS and ICANS are rare events. A significant complication was COVID‐19 infection, leading to therapy discontinuations and deaths. Infection risk varied (6% COVID‐related deaths with odronextamab, 5% with epcoritamab, none with mosunetuzumab) [28, 29, 30, 31], confounded by the trial timing during the COVID‐19 pandemic. A meta‐analysis of BsAb trials in FL and LBCL reported grade 3+ infection rates at 21% and fatal infections at 3% in FL [36]. Notably, 32% of infection‐related deaths lacked a reported cause. Fixed‐duration BsAb regimens are increasingly favored to reduce infection risk, though pivotal trials suggest infections mainly occur in the first 6–12 months of therapy [29, 30]. Ongoing Phase 3 trials will be instrumental in clarifying the infection risk relative to chemotherapy, while real‐world data will assess these risks in non‐trial populations. Ongoing combination studies will shed further light on the infection susceptibility, particularly together with other B‐cell depleting therapies [37, 38, 39].

### 3.3 New Options Beyond CAR‐T and BsAbs

Licensed first relapse options remain limited and suboptimal for POD24 patients, making 2L CAR‐T and BsAb trials highly anticipated. Non‐cross‐resistant immunochemotherapy is often used, especially for prolonged first remissions or planned autologous transplant consolidation. R2 (12‐month regimen) is a well‐tolerated, chemotherapy‐free option in 2L+ and suitable for frailer patients [14]. The use of ASCT and allogeneic transplant is diminishing, reserved for select patient populations.

Loncastuximab tesirine (CD19‐directed ADC) shows promising activity in 2L+ R/R FL (CR 67%, manageable toxicity in a small Phase 2 cohort) [40]. Adding tafasitamab, an anti‐CD19 antibody, to R2 in R/R FL patients improved PFS by 57% in the inMIND trial after a median follow‐up of 14 months [41]. Bruton tyrosine kinase (BTK) inhibition as monotherapy has had disappointing efficacy in FL compared to other B‐cell lymphomas [42, 43]. However, the ROSEWOOD trial showed obinutuzumab‐zanubrutinib was superior to Obinutuzumab alone (ORR/CR: 69%/39% vs. 46%/19%, median PFS: 28 vs. 10.4 months), leading to US and EU approvals [44]. The ongoing MAHOGANY Phase 3 study is evaluating this combination against R2 in 2L+ FL (NCT05100862).

### 3.4 Considerations for Sequencing in R/R FL

Choosing between therapies remains complex due to limited randomized studies, necessitating indirect comparisons. Treatment choices should be guided by several factors, including prior therapy (type, quality, response duration), symptoms, patient fitness, impact on quality of life, and therapy availability and costs—an area of growing disparity across geographical regions.

While T cell‐engaging therapies set a high efficacy benchmark, alternative agents may offer advantages in toxicity, cost, and convenience. Where both CAR‐T and BsAbs are available in 3L+ FL, selection is challenging as no head‐to‐head trials exist. Indirect comparisons suggest CAR‐T may offer superior single‐agent efficacy [45]. While a “one‐and‐done” CAR‐T therapy is appealing, factors such as manufacture times, financial toxicity, need for specialist centers and both short‐ and long‐term toxicities must be considered (e.g., 27% and 34% requiring immunoglobulin replacement post‐CAR‐T in ZUMA‐5 and ELARA) [23, 24, 25, 26]. On the other hand, BsAbs offer an off‐the‐shelf option with rapid initiation, lower CRS/ICANS, and outpatient feasibility. These advantages, along with their combinability, may facilitate earlier use in the treatment sequence. Emerging data suggest efficacy in 1 and 2L settings, including combinations with lenalidomide +/− rituximab or chemotherapy [38, 39].

The impact of extended lymphodepletion due to prior bendamustine on sequencing might be important. Though small numbers, patients with bendamustine exposure 6–12 months before axi‐cel experienced poor outcomes [24], mirroring LBCL findings [46]. Recent BsAb data indicate no impact from bendamustine within 24 months [47], but further studies are needed for closer temporal exposures.

BsAb treatment may increase CD20 antigen loss compared to monoclonal antibodies, with 34% CD20‐negative escape reported post‐mosunetuzumab [48]. This raises concerns about earlier BsAb use, though CD19‐directed salvage options provide reassurance. Long‐term real‐world data and safety reporting remain crucial for identifying rare but serious adverse events in these transformative therapies.

## 4 All Roads to Precision‐Approaches: Future or Fallacy?

### 4.1 Upfront Prognostication

Identifying high‐ and low‐risk FL patients at diagnosis to guide treatment has been a longstanding goal. Clinical indices (FLIPI, FLIPI2, PRIMA‐PI, and FLEX) [49, 50, 51, 52], biological tools (m7‐FLIPI, a clinico‐genetic model, gene expression‐based PRIMA‐23) [53, 54], and baseline FDG PET‐CT metrics (SUV_max_, total metabolic tumor volume) [55, 56] enable upfront risk stratification. FLIPI remains the most used in clinical practice and trials, due to its simplicity and predictive performance. Indeed, a comparison of FLIPI, FLIPI‐2, PRIMA‐PI, and m7‐FLIPI in R‐chemotherapy‐treated patients found FLIPI to be the most effective in predicting PFS and OS [57]. However, it lacks the high prognostic accuracy required for an upfront risk‐adapted approach, with the only clinical implication being the preference for obinutuzumab over rituximab in intermediate‐high‐risk FLIPI patients, based on GALLIUM data.

While FL's genetic landscape is well‐characterized, individual gene mutations rarely show clear prognostic significance. Emerging biological subgroups, defined by genetic subtypes or T‐cell subset composition, refine FL classification and differentiate clinical behavior [58, 59, 60]. The next step is validating these classification systems and assessing whether specific biological subgroups respond preferentially, or not, to existing therapies.

### 4.2 Dynamic Response Assessment and Response‐Adapted Approaches

Given the challenges with upfront prognostication, later timepoints are likely more amenable to defining prognosis and response‐adapting accordingly.

End of induction (EOI) PET‐CT strongly predicts PFS and OS in R‐chemo and O‐chemo‐treated patients [61]. In the GALLIUM study, EOI complete metabolic response (CMR) was the only independent predictor of outcome, with 5‐year PFS of 70.0% for CMR versus 24.9% for non‐CMR, and 5‐year OS of 92.0% versus 79.6% [61].

MRD status following immunochemotherapy has long been associated with outcomes, even after R2 induction in the RELEVANCE study [62, 63]. In GALLIUM, mid‐induction and EOI MRD positivity predicted poorer PFS. Dynamic MRD monitoring was highly informative, with early MRD negativity experiencing with superior PFS while MRD reappearance during maintenance was linked to subsequent relapse [13]. Combined PET‐CT and MRD assessment further refined risk stratification, with MRD‐negative, EOT PET CMR patients showing a 3‐year PFS of 81%.

The FOLL12 study explored post‐induction MRD‐directed therapy adaptation, randomizing PR/CMR patients after R‐chemo to either standard maintenance or an experimental arm using MRD‐directed rituximab or escalation to radio‐immunotherapy for PR cases [64]. The study was negative, showing worse PFS in the experimental arm (3‐year PFS 86% vs. 72%), but it demonstrated the feasibility of real‐time molecular response‐guided therapy. MRD tracking has historically been hindered by the 25%–40% of patients without a PCR‐trackable marker, as in the FOLL12 study. NGS‐based ctDNA assays in FL offer promise in increasing MRD applicability and perhaps sensitivity and specificity [65].

Ongoing trials such as PETReA and FOLL19 aim to further evaluate response‐adapted therapy guided by PET‐CT. PETReA randomizes EOI CMR patients to maintenance or no maintenance anti‐CD20, and PET‐positive patients to standard or intensified therapy with lenalidomide [66]. FOLL19, a follow‐up to FOLL12, retains maintenance anti‐CD20 for all but examines whether reducing induction cycles from six to four is feasible for early CMR and MRD‐negative achievers (EudraCT: 2020‐003277‐22).

Emerging data and technological advances are shaping a toolkit to optimize efficacy while minimizing toxicity, duration, and costs. We strongly advocate for MRD monitoring to be incorporated as a mandatory correlative together with imaging in all future FL trials to examine dynamic response assessments and its potential for biomarker‐guided therapeutic selection.

### 4.3 Predictive Biomarkers

The superior response of EZH2‐mutant FL to EZH2 inhibitor, tazemetostat (ORR 69%/CR 13%) compared to wildtype FL (ORR 34%/CR 4%) exemplifies the potential of biomarker‐driven precision therapies in FL [67]. Yet, its impact on PFS (14 vs. 11 months) remains modest, leading to FDA approval irrespective of EZH2 status.

As therapeutic options continue to expand, the need to match the right drug to the right patient becomes even more critical. Predictive biomarkers are one cornerstone to this goal but remain underdeveloped in FL. Accelerating their discovery requires leveraging both retrospective cohorts and prospective studies to achieve optimal sample sizes. Standardized methodologies, strategic sample acquisition (tissue and blood), and rigorous validation are essential to advancing this effort [68].

## 5 Conclusions

Progress is being made across all clinical stages of FL. With the growing array of treatment options and as patient outcomes improve and treatment goals evolve, optimizing therapy selection and sequencing remains a challenge. Bridging the translational gap requires rigorous preclinical research and the development of precise, yet affordable and accessible, molecular and imaging biomarkers to guide personalized treatment decisions.

## Conflicts of Interest

J.O. has received research funding from AstraZeneca, BeiGene and Genmab and reports consultancy for AstraZeneca, Genmab and Incyte Corporation.

## Peer Review

The peer review history for this article is available at https://www.webofscience.com/api/gateway/wos/peer-review/10.1002/hon.70070.

## Data Availability

The authors have nothing to report.
